# Ultra-sensitive, graphene metasurface sensor integrated with the nonradiative anapole mode for detecting and differentiating two preservatives

**DOI:** 10.1515/nanoph-2024-0126

**Published:** 2024-07-11

**Authors:** Gui Fang Wu, Feng Ping Yan, Xin Yan, Wei Wang, Ting Li, Zhen Hua Li, Lan Ju Liang, Rui Zhang, Fu Tong Chu, Hai Yun Yao, Meng Wang, Zi Qun Wang, Lu Wang, Xiao Fei Hu

**Affiliations:** School of Electronic and Information Engineering, 47829 Beijing Jiaotong University, Beijing 100044, China; School of Opto-Electronic Engineering, Zaozhuang University, Zaozhuang 277160, China; School of Physical Science and Engineering, 47829 Beijing Jiaotong University, Beijing 100044, China; School of Electrical and Information Engineering, Anhui University of Science and Technology, Huainan, Anhui 232001, China; School of Semiconductor and Physics, North University of China, Taiyuan 030051, China

**Keywords:** metasurface, nonradiative anapole, multidimensional sensing, graphene

## Abstract

Graphene-based metamaterial sensors are of significant research value for detecting food preservatives at low concentrations due to their extremely high sensitivity levels. In this work, we proposed and experimentally demonstrated an anapole resonance-based graphene metasurface (An-graphene-Ms) sensor with its conductivity altered by electrostatic doping effects for detecting and differentiating between two preservatives, sodium benzoate and potassium sorbate, in the terahertz region. Sodium benzoate, owing to its benzene ring structure, established *π*–*π* stacking interactions between the *π*-electrons in the benzene ring and those in graphene, amplifying the sensing effect. The amplitude changes and phase differences of the An-graphene-Ms sensor for the sodium benzoate detection were greater than those for potassium sorbate at the same concentration. Additionally, to reveal the dependence of the resonance frequency on the time delay, the measured signals were investigated using the continuous wavelet transform (CWT), and the time-frequency combination of the metasurface sensor was performed. The 2D wavelet coefficient intensity cards are effectively constructed through CWT, which also presents a more accurate approach for distinguishing and determining the concentrations of the two preservatives.

## 1 Introduction

Adding food preservatives during food production and processing has increased dramatically. Sodium benzoate and potassium sorbate are the most commonly used food preservatives [1]. Nevertheless, excessive access to preservatives probably increases the probability of suffering from asthma, kidney failure, stomach trouble, and cancer [2]. Compared to sodium benzoate, potassium sorbate has a better preservative effect and less toxicity; hence, it has become a better substitute for sodium benzoate [3]. The standard for using food additives implemented in China (GB 2760-2014) clearly states that the maximum amount of both preservatives added to food should not exceed 2 g/kg. Some countries in the European Union also have strict standards for the amount of these two food additives added. Hence, the researchers attached great importance to detecting the content of these two preservatives [4]. Gas chromatography, high-performance liquid chromatography, and other methods are commonly used to detect sodium benzoate and potassium sorbate with simple operation and high accuracy. However, the disadvantages are the requirements of complicated pretreatment of the analytes and the inability to detect the lower concentration of the analytes. Thus, there is an urgent need for a method that can rapidly detect and differentiate between sodium benzoate and potassium sorbate at low concentrations.

Terahertz (THz) waves have low energy and fingerprint properties and can provide a fast and nondestructive detection method [5], [6], [7], [8], [9], [10], [11]. Yet, the detection sensitivity is limited by the mismatch between the size of the target analyte and the THz wavelength [12], [13], [14], [15]. The combined application of metasurface and THz time-domain spectroscopy techniques has received much attention in the field of sensing and detection to improve detection sensitivity [16], [17], [18], [19], [20], [21]. Particularly, the nonradiative anapole mode can further enhance the nonlinear electromagnetic properties of metasurfaces, which is a unique optical state induced by the interference of electric dipole (ED) and toroidal dipole (TD) moments. The anapole mode confines the electromagnetic field inside the scattering source, like the metamaterial unit structure. So, the energy is not radiated to the outside and usually has a very high quality factor [22]. In addition, the nonradiative anapole mode can further enhance the nonlinear electromagnetic properties of metasurfaces. The defining nonradiating condition of the anapole relies on the fine balance between the constituent ED and TD excitations. Consequently, such excitations are expected to be highly sensitive to external perturbations including variations in the ambient refractive index [23], [24]. Compared with conventional dipole modes with low transmission, anapole modes can acquire the effective transmission channel due to the nonradiative property, which is beneficial to practical low-loss sensors [25]. Thus, the nonradiative anapole mode can further improve the sensitivity of the metamaterial sensor in detecting analytes. Nevertheless, researchers found that the THz metasurface sensor based on anapole resonance is still insufficient to detect low analyte concentrations. Furthermore, it is necessary to enhance the interaction between THz waves and analytes by other methods or materials [26]. Graphene, which consists of a single layer of carbon atoms, is one of the hotspots in research on metamaterials. This is due to its near-perfect optoelectronic properties, such as ultra-low transmission loss and extremely high carrier mobility. Consequently, researchers have gradually proposed various graphene-based metamaterial sensors and modulators since the Fermi energy level (*E*_F_) and charge carrier density of graphene can be tuned optically, electrically, and chemically. Combining graphene and metasurfaces with anapole resonance provided a well-developed basis for detecting femtogram-level concentrations in chemical and biological sensing. Besides, graphene possesses a *π*-electron structure on its benzene ring, allowing for noncovalent bonding interactions named *π*–*π* stacking between aromatic rings, modifying the internal electron band structure of graphene. This doping effect changes with charge concentration and affects conductivity, resulting in the electronic and optical modification of the graphene layer [27], [28], [29], [30], [31], [32], [33], [34], [35]. Then, the THz graphene-based metasurface sensors may show enhanced sensitivity in detecting analytes containing aromatic rings due to *π*–*π* stacking effects. Guo et al. [34] developed a sensitive label-free electrochemical sensor to detect thrombin and lysozyme. An aromatic dye, Orange II, was chosen for the noncovalent functionalization of chemically reduced graphene, which is a negatively charged water-soluble molecule with a large planar aromatic surface. It strongly interacted with graphene through *π*–*π* stacking, and the sensor demonstrated a high sensitivity property. Then, Xu et al. [35] proposed an efficient and label-free THz graphene metamaterial sensor, which successfully detected the existence of chlorpyrifos-methyl molecules, and the modulation depth reached 35 %. It was also shown that substances interacting with p-electrons, such as chlorpyrifos-methyl and graphene, showed higher sensitivity through *π*–*π* stacking than substances without *π*-electrons (fructose), and the detection limit of chlorpyrifos-methyl could be reduced to 0.2 ng. Our team successfully investigated that due to graphene incorporation and the *π*–*π* stacking effect, the detection limits for tyrosine and arginine were 26.43 and 581.4 fg/mL, respectively. These reported graphene metasurface sensors were verified based on amplitude changes due to the *π*–*π* stacking effect of graphene with analytes containing aromatic rings can improve the sensitivity of the sensors, but sensors for detecting and differentiating between the two samples at femtogram-level concentrations through phase differences, amplitude changes, and time-frequency association have not been reported.

Here, we proposed and experimentally demonstrated an anapole resonance-based graphene metasurface (An-graphene-Ms) sensor for detecting and distinguishing two preservatives, sodium benzoate and potassium sorbate. First, we investigated the physical mechanism of anapole resonance generated by the proposed metasurface by comparing the electromagnetic responses of ED and TD moments. The superior characteristics of the anapole mode perfectly matched the requirements of highly sensitive sensing applications. In addition, the ability of the proposed sensors with and without graphene to detect two preservatives was compared. The sensors containing graphene showed excellent sensitivity characteristics. Then, the experimental results showed that analytes with p-electrons, like sodium benzoate, which interacted with graphene through *π*–*π* stacking, had a higher sensitivity than analytes without *π*-electrons, such as potassium sorbate. Finally, we detected and differentiated the two preservatives rapidly at low concentrations. It was based on the large difference in the detection limits of the two preservatives by the An-graphene-Ms sensor, the amplitude change (∆*T*) and the phase difference (∆*P*) of the sensor for detecting sodium benzoate were greater than that for detecting potassium sorbate at the same concentration. And, the 2D wavelet coefficient intensity cards are effectively constructed through continuous wavelet transforms, which also presents a more accurate approach for distinguishing and determining the solution concentrations of the two preservatives.

## 2 Materials and methods

**Figure 1:**
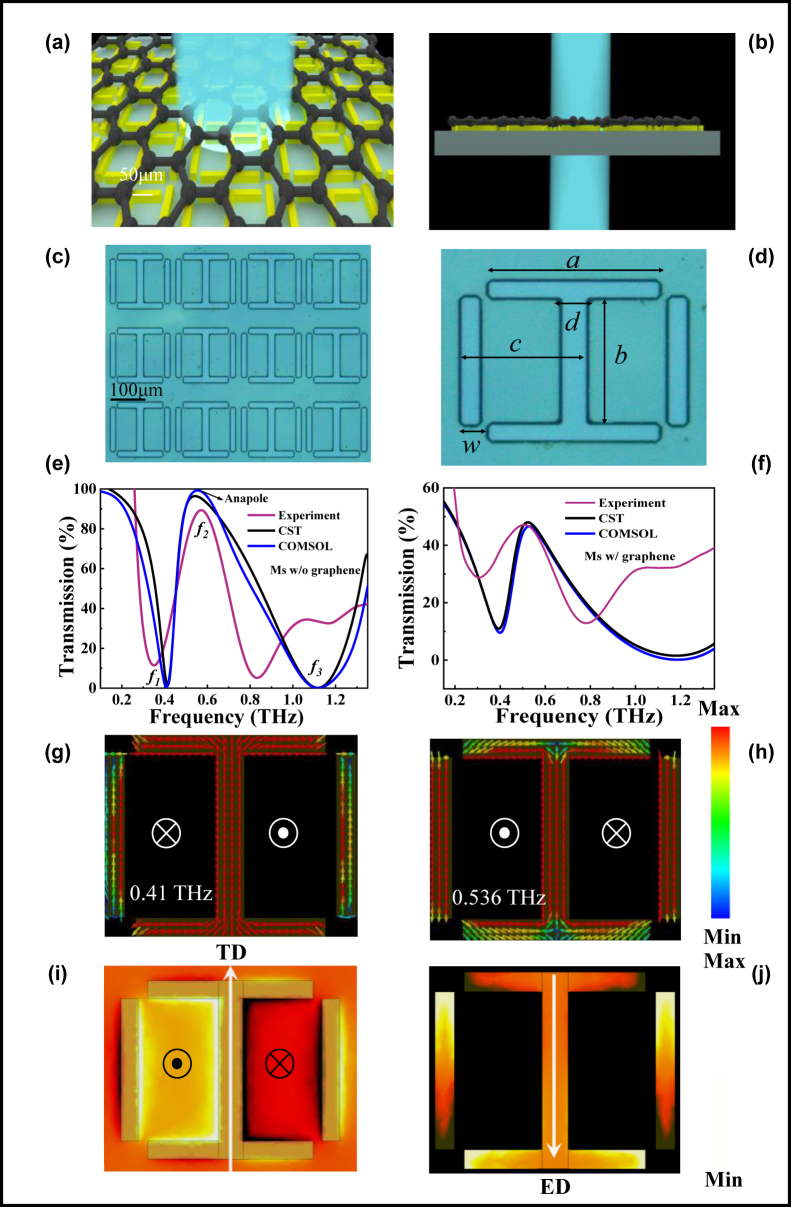
Experimental design of the proposed graphene metasurface sensor. (a) An artistic sketch of the proposed terahertz sensor integrating a graphene layer, an aluminum metal layer, and a polyimide substrate. (b) A side view of the proposed THz sensor. Different colors denote different constituent materials. (c) A microscopic image of the metamaterial array under an optical microscope. (d) A top view of the unit cell of the proposed metasurface; the geometric parameters of the unit cell: *a* = 140 μm, *b* = 110 μm, *c* = 89.75 μm, *d* = 20 μm, and *w* = 15 μm. (e) The transmission curves of AnMs well matched under experimental and simulated conditions. (f) The transmission spectra of An-graphene-Ms obtained by experiments and simulations. (g) The surface current distribution of the AnMs at 0.41 THz. (h) The simulated surface current distribution at 0.536 THz. (i) The AnMs formed a toroidal dipole moment along the +*y*-axis. (j) The AnMs produced an electric dipole moment along the −*y*-axis.

Figure 1(a) and (b) shows the top and side views of the three-dimensional (3D) structure of the proposed An-graphene-Ms sensor, respectively. The sensor consisted of a graphene layer at the top, a metal layer in the middle, and polyimide as the substrate. Figure 1(c) and (d) shows the optical microscope image of the anapole resonance-based metasurface (AnMs). It can be seen that the uniformity of the array structure was excellent. The aluminum structural unit consisted of an I-shaped resonator and two cut-wire resonators, and its period was 205 μm. The fabrication details are provided in Figure S1 in the Supporting Information. In this work, all numerical simulations were conducted simultaneously with the frequency solvers based on CST and COMSOL to verify the reliability of the simulation data. A THz wave was incident vertically on the upper surface of the sensor, with the electric field along the *y*-direction (*E*_*y*_) and the magnetic field along the *x*-direction (*H*_*x*_) (the effect of the angle of incidence of terahertz waves on the transmission curve is provided in Figure S2 in the Supporting Information). As presented in Figure 1(e), the transmission spectrum of the metasurface was calculated in the 0–1.35 THz frequency range. The frequencies of the peak and trough were 0.41 and 0.536 THz, respectively. Figure 1(f) shows the transmission spectra of the An-graphene-Ms under experimental and simulated conditions. There was a large radiation loss due to the higher carrier concentration and conductivity of graphene, which attenuated the amplitude of the transmission curve with an insignificant frequency shift. The proposed metasurfaces with or without graphene well matched the transmission curves obtained by experiments and two simulation software. These slight differences can be attributed to errors in fabricating the polyimide and metal structure. The corresponding distributions of the surface current in the *x*–*y* plane at frequencies of 0.41 and 0.536 THz are illustrated in Figure 1(g) and (h), respectively, to better analyze the mechanism of the resonance generated by the proposed AnMs. At 0.41 THz, the left rectangle of the aluminum metal unit formed a clockwise circular current that produced a magnetic dipole (MD) moment along the −*z*-axis. Similarly, the right rectangle produced an MD along the +*z*-axis. This pair of opposite MD moments was connected head-to-tail to form a TD along the −*y* axis. At 0.536 THz, the left and right rectangular currents formed +*z*-directed and −*z*-directed MDs, respectively, and this pair of MD moments in opposite directions formed a TD along the +*y*-axis, as shown in Figure 1(i). This is because the difference in oscillating charge accumulated in the upper and lower parts of the I-shaped resonator produced an ED moment along the −*y*-axis, as shown in Figure 1(j). Hence, the interaction of the proposed metasurface with THz waves was mainly characterized by ED and TD resonance.

**Figure 2:**
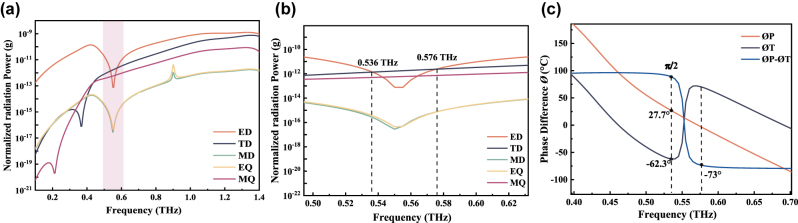
Anapole metadevice features. (a) The far-field scattering power of different multipoles. (b) The locally magnified image showing the same scattered power for ED and TD at 0.536 and 0.576 THz. (c) *P* and *T* were in opposite phases with a phase difference of *π*/2 at 0.536 THz, while the phase difference between *P* and *T* at 0.576 THz did not satisfy the conditions for anapole resonance excitation.

As shown in Figure 2(a), the scattering powers of different multipoles were calculated through the spatial current density distribution based on the Cartesian coordinates [36], [37], [38]. The electric quadrupole (EQ), magnetic quadrupole (MQ), TD, ED, and MD are the five highest contributors, and the octopoles and higher-order multipoles can be ignored due to their extremely weak influence on the scattered power. At 0.41 THz, the ED resonance generated at the metasurface dominated, and the far-field power of the ED reached a maximum. At 0.536 and 0.576 THz, ED and TD contribute equally to the far-field scattering power and both dominate, as shown in Figure 2(b). Hence, we further analyzed the phases of *P* and *T* (i.e., Ø*P* and Ø*T*), as well as the phase difference (Ø*P* – Ø*T*), as indicated in Figure 2(c). At 0.536 THz, *P* and *T* were in opposite phases with a phase difference of *π*/2 (i.e., *P* = *ikT*) [36], [37], [38], such that TD and ED produced a destructive interference, leading to the complete disappearance of far-field radiation and a significant enhancement of near-field localization. In contrast, the phase difference between *P* and *T* at 0.576 THz did not satisfy the conditions for anapole resonance excitation. It should also be noted that the total scattering was not completely suppressed even in the anapole mode, and the total far-field scattering of the sample was not zero, with a transmission coefficient of less than 100 % at 0.536 THz, as shown in Figure 1(e). This is because the far-field scattering caused by the dominant ED and TD was canceled, but the far-field radiation produced by the other multipoles (especially the MQ) was excited, leading to nonzero extinction.

## 3 Results and discussion

The AnMs sensor and the An-graphene-Ms sensor were used as detection platforms for two common preservatives. Sodium benzoate and potassium sorbate were detected at the same concentration, as indicated in Table 1, to facilitate the comparison of the proposed sensors to detect any difference in the performance of the two preservatives. The sample preparation and measurement methods are provided in the Supporting Information.

**Table 1:** The specific concentrations of sodium benzoate and potassium sorbate detected in the experiment.

C_1_	C_2_	C_3_	C_4_	C_5_	C_6_	C_7_	C_8_
0.12 fg/mL	0.24 fg/mL	0.36 fg/mL	5.48 fg/mL	15.72 fg/mL	0.23 pg/mL	0.65 pg/mL	0.86 pg/mL
**C_9_**	**C_10_**	**C_11_**	**C_12_**	**C_13_**	**C_14_**	**C_15_**	**C_16_**
9.49 pg/mL	26.75 pg/mL	0.38 ng/mL	1.09 ng/mL	15.59 ng/mL	0.61 μg/mL	50.01 μg/mL	1.05 mg/mL

**Figure 3:**
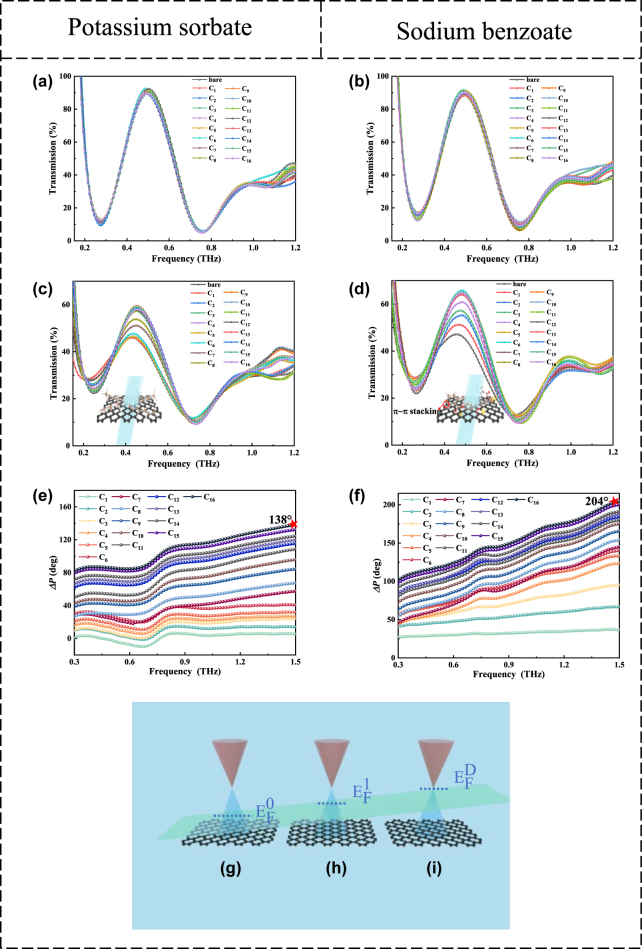
The transmissions and phase spectra of potassium sorbate and sodium benzoate were detected by the proposed sensors. (a) As the potassium sorbate concentration increased, the change in the transmission curve of the AnMs sensor was not significant. (b) The transmission curve of the AnMs sensor also did not change with increasing the sodium benzoate concentration. (c) Transmission curves of An-graphene-Ms sensor with increasing the potassium sorbate concentration. The inset shows no interaction between potassium sorbate molecules and graphene. (d) As the sodium benzoate concentration increased, the transmission curve of the An-graphene-Ms sensor changed significantly. The inset shows that the sodium benzoate molecules possessed a benzene ring structure with *π*-electrons, facilitating enhanced sensing capabilities through strong interaction with the *π*-electrons of graphene by *π*–*π* stacking. Phase difference Δ*P* was calculated between the bare sample and the concentration of C_1_–C_16_, (e) sodium benzoate, (f) potassium sorbate. (g)–(i) *E*_F_ variations of graphene at different concentrations. The *E*_F_ of graphene shifted asymptotically from the valence band to the Dirac point.

Figure 3(a) and (b) shows the transmission spectra of the AnMs sensor detecting an increase in the analyte concentration from C_1_ to C_16_. The transmission spectra at all concentrations did not differ significantly from those with no analyte (bare). The results show that the AnMs sensor could not detect trace concentrations of two preservatives. Figure 3(c) shows the transmission spectrum of the An-graphene-Ms sensor detecting potassium sorbate. As the concentration increased from C_1_ to C_5_ (15.72 fg/mL), the transmission coefficient did not differ significantly from those with no potassium sorbate. Next, as the concentration increased from C_5_ to C_9_ (9.49 pg/mL), the maximum value of the transmission coefficient of the An-graphene-Ms sensor increased linearly; when C_9_ increased to C_16_ (1.05 mg/mL), the maximum value of the transmission coefficient appeared to be saturated. The transmittance curves of the An-graphene-Ms sensor for the detection of sodium benzoate were quite different from those of potassium sorbate; as the concentration increased from C_1_ to C_5_ (15.72 fg/mL), the maximum transmission coefficient value of the An-graphene-Ms sensor increased linearly, and the detection limit for sodium benzoate was C_1_ (0.12 fg/mL). When C_5_ increased to C_16_ (1.05 mg/mL), the maximum transmission coefficient value appeared to be saturated, as illustrated in Figure 3(d) (the detailed analysis of why this saturation phenomenon occurs can be found in Figure S3 in the Supporting Information). The predominant sensing mechanism is probably that the preservatives-induced electrostatic doping effect may also cause changes in the conductivity of the graphene layer by changing its charge polarity and carrier concentration. This effect occurs when the free electron/hole charges induced by electrostatic field excitation replaces the charge normally provided by the donor/acceptor species. And, the graphene is usually p-doped, so the initial Fermi energy level (*E*_F_) of graphene is slightly out of the Dirac point and in the valence band (see Figure 3(g)). To move *E*_F_ from the valence band to the Dirac point requires a very small external stimulus, so preservative solutions can be successfully detected at femtogram level concentrations based on this principle. As the concentration of sodium benzoate increases, the *E*_F_ gradually shifted from the valence band to the Dirac point (see Figure 3(h)). As the concentration increased to C_6_ (0.23 pg/mL), the *E*_F_ shifted to the Dirac point (see Figure 3(i)). Since when the *E*_F_ of graphene is closer to the Dirac point, the conductivity of graphene is lower, the loss would also be lower [29], [31], [35]. Thus, the transmission coefficient reached its maximum at C_6_. When the concentration continued to increase, the *E*_F_ was still near the Dirac point, and the transmission coefficient no longer changed significantly. The transmission coefficient variation of the sensor at different concentrations, C_1_–C_16_, is defined as ∆*T* = (*T*_Cc_ *– T*_Bare_) %, where *T*_Cc_ (*T*_Bare_) is the transmission at the resonance point with (without) the analyte. The maximum ∆*T* was reached at a concentration of 0.23 pg/mL (C_6_) of sodium benzoate, where ∆*T*_max_ = 35.52 %, enabling ultra-sensitive detection. Simultaneously, the electromagnetic properties, and therefore the effective resonant characteristics of the metamaterial unit, can be adjusted via controlling the distribution of the carriers of the An-graphene-Ms to modulate the phase of the perpendicular incident THz waves. In addition, as shown in Figure 3(e) and (f), the higher the frequency, the stronger the phase modulation effect of the analyte on this sensor. One possible explanation is that the local field at higher frequencies is more sensitive to changes in the dielectric environment because of mode coupling. To characterize the sensing performance quantitatively, Δ*P* = *P*_0_ − *P*_*i*_ (*P*_0_ and *P*_*i*_ are values of phases at C_0_ and C_*i*_, respectively) were calculated between the bare sample and the concentration of C_1_–C_16_, as illustrated in Figure 3(e) and (f). The phase difference at the same frequency point basically increases with the increase of the concentration of the two preservatives, and the maximum phase difference of the sensor is 138° and 204° when the concentration of potassium sorbate and sodium benzoate is 1.05 mg/mL (C_16_), respectively. This device realizes ultrasensitive multidimensional dynamic sensing of the two preservatives.

Generally, we utilize the Fast Fourier Transform (FFT) to extract the frequency components for analysis. However, this method comes at the expense of losing information about the time domain, which is extremely disadvantageous for metasurface-based sensors in the THz band. For a deeper understanding of the information provided by the metasurface sensors, we experimentally propose a new signal processing method that uses the continuous wavelet transform (CWT) in both the time and frequency domains. This combination of analyses allowed for a comprehensible description of the signal characteristics. For test signals in time domain, the CWT is an algorithm that compares a test signal *f*(*t*) with a wavelet function and its essential definition can be expressed as follows [39], [40], [41], [42]:

(1) $$W_{\psi }f\left(a,b\right)=\overset{\infty }{\underset{-\infty }{\int }}f\left(t\right)\frac{1}{\sqrt{a}}\psi^{*}\left(\frac{t-b}{a}\right)dt$$

where *a* is a scale parameter analogous to frequency, *b* is a position parameter analogous to time positional parameter of the time delay, and Ψ^∗^ denotes the complex conjugate, which equation can be expressed as [41]:

(2) $$\begin{array}{c} \psi_{a,b}\left(t\right)=\psi_{a}\left(t-b\right)=\frac{1}{\sqrt{a}}\psi \left(\frac{t-b}{a}\right) \end{array}$$

(3) $$\begin{array}{c} W_{\psi }f\left(a,b\right)=\left\langle f\left(t\right),\psi_{a,b}\left(t\right)\right\rangle \end{array}$$

**Figure 4:**
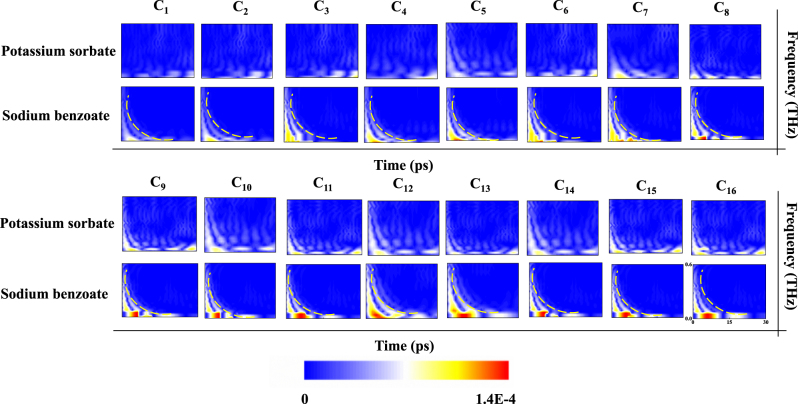
The 2D extinction intensity plots of the two preservatives at each concentration can be used as standard graphic cards to differentiate between them as well as the solution concentrations.

The inner product of the wavelet function and the time domain signal could be acquired by resolving Eq. (3). By this method, it is possible to convert one-dimensional information into a two-dimensional function with two variable parameters: the scale parameter *a* and the position *b*. Wavelet coefficients (WCs) with different concentrations can be obtained depending on the values of *a* and *b*. The complex Morlet function is selected as mother wavelet [40], [41]:

(4) $$\psi \left(t\right)=\frac{1}{\sqrt{\pi f_{b}}}\cdot e^{j2\pi f_{c}t}-\left(t^{2}/f_{b}\right)$$

where *f*_*c*_ and *f*_*b*_ denote the central frequency and bandwidth of the Morlet wavelet, both selected as 3 THz. We acquired the time signals by THz time-domain spectroscopy and used Eqs. (3) and (4) to obtain the joint time-frequency two-dimensional signals. By processing the time signals of different concentrations of the two preservatives, we acquired 2D wavelet coefficient (WC) intensity cards for the corresponding concentrations, as illustrated in Figure 4. Graphical depictions including detailed information about THz waves and interactions between the two preservatives can be used as standard graphic cards to distinguish between them as well as solution concentrations. In particular, the intensity of the WC increased significantly when the concentration of the preservative was increased from C_1_ to C_16_. Similar to the mechanism of amplitude change mentioned above: the change in solution concentration leads to a change in the *E*_F_ of graphene, which affects the strength of the interaction between the THz wave and the analyte and leads to a change in the strength of the resonance of the anapole and the WCs. Furthermore, it is observed from Figure 4 that the time-frequency distributions of extinction intensities of potassium sorbate and sodium benzoate are completely different. The An-graphene-Ms sensor detects sodium benzoate with a relatively strong intensity of its interaction with terahertz waves, which is concentrated near the 2 and 10 ps time points in the time domain and near the 0 and 0.5 THz frequencies in the frequency domain. In contrast, the extinction intensity of potassium sorbate splits discrete shapes, involving both the 0 and 30 ps time ranges in the time domain. Next, the hot zone of sodium benzoate shows a “moon” shape, and at the same concentration, the extinction intensity is significantly stronger than that of potassium sorbate. These results indicate that the measured 2D wavelet coefficient intensity cards can be used to quickly differentiate between the two preservatives and their solution concentrations by comparing them to the corresponding standard cards.

**Figure 5:**
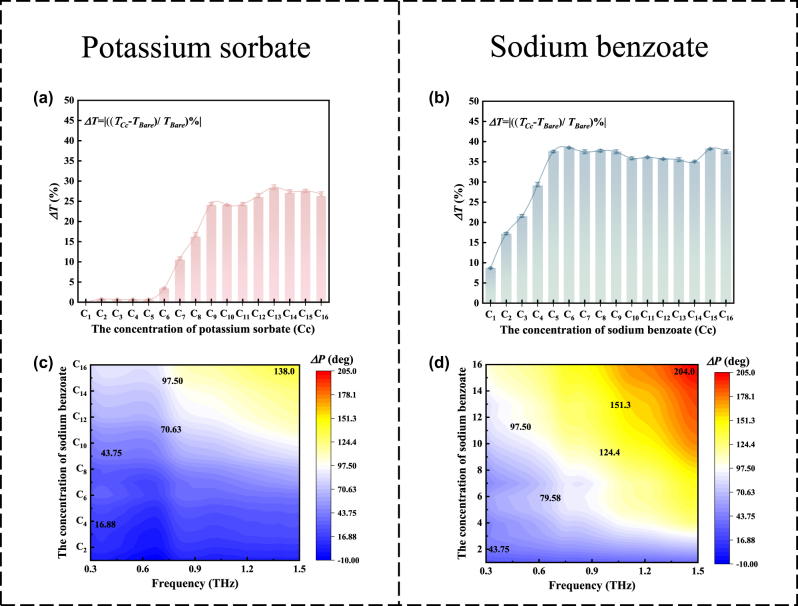
Quantitative analysis of sensing performance. (a) Amplitude changes of the An-graphene-Ms sensor at different concentrations of potassium sorbate and ∆*T*_max_ = 28.45 %. (b) Amplitude changes of the An-graphene-Ms sensor at different sodium benzoate concentrations and ∆*T*_max_ = 38.52 %. Phase change of two preservatives at each concentration, (c) potassium sorbate, (d) sodium benzoate.

Notably, the An-graphene-Ms sensor enabled trace detection of two preservatives and could be used to differentiate between sodium benzoate and potassium sorbate in four different ways: (1) the sodium benzoate detection limit of this sensor was C_1_ (detection limit = 0.12 fg/mL), far more than that of potassium sorbate (detection limit = 0.23 pg/mL); (2) the ∆*T*_max_ and ∆*P*_max_ of sodium benzoate detected by this sensor were 38.52 % and 204°, respectively, which are greater than that of potassium sorbate (∆*T*_max_ = 28.45 %, ∆*P*_max_ = 138°); (3) the ∆*T* and ∆*P* of the sensor for detecting sodium benzoate were greater than that for detecting potassium sorbate at the same concentration, as seen from comparing Figure 5; and (4) the most significant way to distinguish between sodium benzoate and potassium sorbate is that there is a very obvious difference in the two-dimensional extinction cards for different concentrations of sodium benzoate and potassium sorbate. The reason for the different sensing performance of this sensor in detecting the two preservatives may be because the sodium benzoate molecules possessed a benzene ring structure with *π*-electrons. This facilitated enhanced sensing capabilities through strong interaction with the *π*-electrons of graphene by *π*–*π* stacking. More specifically, this doping effect changed with the charge concentration, affecting the conductivity and resulting in the electronic and optical modification of the graphene layer. The *π*–*π* stacking effect promoted the *E*_F_ of graphene toward the Dirac point and decreased the carrier density in graphene [30], [34], [35]. Accordingly, the conductivity of graphene decreased, and the anapole resonance of the metamaterial excitation was enhanced. This led to an increase in the transmittance of the An-graphene-Ms sensor. So, when detecting sodium benzoate at the same detection concentration, the transmittance was much higher than that of potassium sorbate. Thus, the anapole resonance excited by the An-graphene-Ms sensor provided a platform for effective control of far-field radiation and near-field enhancement in optics. This is expected to enable the detection and differentiation of the two preservatives at low concentrations, with better performance than previously reported sensors, as indicated in Table 2.

**Table 2:** Comparing the performance with previous work using THz metasurface sensors.

Sensors	Analytes	LOD	∆*T*	IBEMG
Graphene +Ms [29]	Chlorpyrifos-methyl	0.13 mg/mL	17 %	*π*–*π* stacking
Graphene + Ms [35]	Chlorpyrifos-methyl	0.2 ng/mL	35 %	*π*–*π* stacking
Graphene + Ms [43]	Chlorothalonil	100 pg/mL	15 %	*π*–*π* stacking
Graphene + Ms + perovskite [44]	Whey protein	2.51 μg/mL	37 %	No *π*–*π* stacking
This work	Sodium benzoate	0.12 fg/mL	38.52 %	*π*–*π* stacking
	Potassium sorbate	0.23 pg/mL	28.45 %	No *π*–*π* stacking

LOD: the limit of detection. Δ*T*: the modulation depth. IBEMG: the interaction between external molecules and graphene. Ms: metasurface.

## 4 Conclusions

In this work, we experimentally explored and exhibited a THz An-graphene-Ms sensor for detecting and distinguishing two preservatives in the THz region, sodium benzoate and potassium sorbate. The superior characteristics of the anapole mode perfectly matched the requirements of highly sensitive sensing applications. In addition, sodium benzoate, owing to its benzene ring structure, formed *π*–*π* stacking interactions between the *π*-electrons in the benzene ring and those in graphene, amplifying the sensing effect. Moreover, the 2D WC intensity cards are effectively constructed through continuous wavelet transforms, which also presents a more accurate approach for distinguishing and determining the solution concentrations of the two preservatives. The successful development of the standardized WC intensity card and the combination of ∆*T* and ∆*P* demonstrates our ability to detect and identify potassium sorbate and sodium benzoate at low concentrations efficiently and rapidly. In fact, the biological samples detected in the experiments were single substances after processing. However, there are many types of biological substances in practical applications, and continued research is needed to improve the detection reliability and selective identification. Nevertheless, combining graphene and metasurfaces with anapole resonance also provides a well-developed basis for detecting femtogram-level concentrations in chemical and biological sensing.

## Supplementary Material

Supplementary Material Details
