# Supplementary material for: Ultra-sensitive, graphene metasurface sensor integrated with the nonradiative anapole mode for detecting and differentiating two preservatives
Source: Nanophotonics. 2024 Jul 11;13(20):3793–803. doi: 10.1515/nanoph-2024-0126 (PMC11449421; doi:10.1515/nanoph-2024-0126)
Supplement: Supplementary file 1 — Supplementary Material Details [file j_nanoph-2024-0126_suppl_001.docx]

Gui Fang Wu ^a^, Feng Ping Yan ^a, *^, Xin Yan ^b, *^, Wei Wang ^c^, Ting Li ^c^, Zhen Hua Li ^b, *^, Lan Ju Liang ^b^, Rui Zhang ^d^, Fu Tong Chu ^b^, Hai Yun Yao ^b^, Meng Wang ^b^, Zi Qun Wang ^b^, Lu Wang ^b^, and Xiao Fei Hu ^b^

**Ultra-sensitive, graphene metasurface sensor integrated with the nonradiative anapole mode for detecting and differentiating two** **preservatives**

*^a^School of Electronic and Information Engineering, Beijing Jiaotong University, Beijing 100044, China*

*^b^School of Opto-electronic Engineering, Zaozhuang University, Zaozhuang 277160, China*

*^c^School of Physical Science and Engineering, Beijing Jiaotong University, Beijing 100044, China*

*^d^School of Electrical and Information Engineering, Anhui University of Science and Technology, Huainan, Anhui 232001, China*

**Supporting Information**

1. **Experimental as well as simulation and analysis sections**

The experimental setup mainly consists of a homogenizer (model: G3P-8), a photolithography machine (brand name: ABM), a magnetron sputter coater (brand name: Denton), and a THz time-domain spectrometer (model: TAS7500). The preparation of the metasurface samples was carried out by a conventional photolithography and sputter coating process, and the main processing steps are shown in Fig. S1. The specific steps are as follows:

1. The SiO_2_ substrate was ultrasonically cleaned sequentially with acetone, anhydrous ethanol and deionized water;
2. A polyimide (PI) film was spin-coated on the SiO_2_ substrate;
3. Two layers of photoresist LOR10B and AZ1500 were spin-coated on the polyimide film;
4. UV exposure using a photolithography machine and development using a developer solution;
5. Metal layers were sputter deposited using a magnetron sputterer;
6. The samples were immersed in acetone solution for 10 minutes and the remaining metal and photoresist were washed with deionized water;
7. Finally, a 1.0 cm × 1.0 cm monolayer of graphene was transferred to the top of the Al structural units and baked at 100°C for 30 min, completing the the fabrication of an An-graphene-Ms sensor with good uniformity.

**Fig. S1.** The main processing steps for the proposed metamaterial.

The performed extensive numerical simulations of An-graphene-Ms using computer simulation technology (CST) and COMSOL Multiphysics simulation packages. Perfectly matched layers were used in the top and bottom domains. The proposed An-graphene-Ms was vertically illuminated by a THz wave with an electric field along the *y*-direction (*E_y_*) and a magnetic field along the *x*-direction (*H_x_*). The metamaterial microstructures were made of lossy aluminum metal, and the conductivity was set to be $\sigma_{dc}=3.56\times10^{7}S/m$. Polyimide was used as the flexible substrate, and the dielectric constant and tangent loss were 3.4 and 0.05, respectively. In addition, we experimentally propose a signal processing method that uses the continuous wavelet transform (CWT) in both the time and frequency domains. This combination of analyses allowed for a comprehensible description of the signal characteristics. For test signals in time domain, the CWT is an algorithm that compares a test signal f(t) with a wavelet function. Then, we process and analyze the data obtained from simulation and experiment by Python language and origin software.

1. **Sample preparation and measurement methods**

The standard sodium benzoate and potassium sorbate solutions were purchased from MACKLIN (Shanghai, China) (<https://www.macklin.cn/>). The ultra-pure deionized water was absorbed by a pipetting gun to dilute the standard solution with a concentration of 1 mg/mL to determine the trace content of sodium benzoate and potassium sorbate. Next, the standard solutions were continuously diluted by the ultra-pure deionized water to obtain nine sets of sodium benzoate and potassium sorbate concentrations (1 mg/mL, 24.4 μg/mL, 595 ng/mL, 14.5 ng/mL, 353.09 pg/mL, 8.63 pg/mL, 0.21 pg/mL, 5.12 fg/mL, and 0.12 fg/mL). Then, 25 μL of the analyte with a concentration of 0.12 fg/mL (C_1_) was added to the surface of the sensor. After the water in the solution was completely evaporated, the changes in the transmission curve of the sensor were observed by THz time-domain spectroscopy, and the analyte with a concentration of C_2_ was obtained by continuing to add the analyte with a concentration of 0. 12 fg/mL. If the change in the transmission curve was no longer obvious, then the concentration of the next gradient was added, and the concentration was accumulated to get C_i_. Finally, 16 groups of sodium benzoate and potassium sorbate solutions with different concentrations were obtained. To achieve greater reliability, all experimental data provided in this work are averaged over three tests. Moreover, after completing all the experiments for the first biosensor, we repeated them on another biosensor prepared at the same time, using the same conditions, to prove that the results were reliable and not accidental.

1. **The** **effect of the angle of incidence of terahertz waves on the transmission curve**

The THz wave was incident perpendicularly rather than obliquely to the upper surface of the sensor, with the electric field along the *y*-direction (*E_y_*) and the magnetic field along the *x*-direction (*H_x_*). In practical applications, sensors with wide incidence angle insensitivity can avoid errors introduced by differences in the position of the light and thus, improve detection efficiency. The electromagnetic response of the sensor is usually sensitive to the direction of the incident excitation and measurement errors are likely to occur. The angle of incidence (*θ*) is the angle between the incident THz wave and the normal to the sensor surface. To investigate the effect of *θ* on sensor performance, the absorption characteristic curves for different values of *θ* are plotted in Fig. S2(a). The resonant frequency did not change significantly when *θ* was increased from 0° to 30°, and the resonant position only shifted by 0.0078 GHz when *θ* = 30°. As a result, the sensor exhibits high angular stability at oblique incidence angles between 0° and 30°. In addition, the biosensor device was tested in the experiment using a THz time-domain spectroscopy system. The sample was placed on a sample stage as shown in Fig. S2(b), and the THz wave was incident vertically on the surface of the sample. The angle of incidence may be slightly off, but due to the high stability and accuracy of the system's testing, the error in angle is always kept within the normal range.

**Fig. S2.** (a) The effect of the angle of incidence of terahertz waves on the transmission curve. (b) Schematic diagram of the optical path of the test samples in the experiment.

1. **The reason for the saturation phenomenon of the transmittance.**

We experimentally measured the refractive index of potassium sorbate and sodium benzoate to be around 1.6, as presented in Fig. S3(a). So, the simulated analyte with a refractive index of 1.6 was deposited on the surface of this sensor, and the relationship between the terahertz spectra and the thickness of the analyte is shown in Fig. S3(b). As the analyte thickness increased from 0 (bare) to 26 μm, the amplitude change ∆*T* increased until it saturated. This shows that ∆*T* as a function of analyte thickness satisfied the saturation nonlinearity, as indicated in Fig. S3(c). The saturation phenomena appearing in the simulation and experiment were similar. This is mainly because the E_F_ of graphene first shifted from the valence band to the Dirac point with the increase in the concentration (or thickness) of the analyte; the conductivity first decreased and then became gradually unchanged, and the saturation phenomenon gradually occurred after the transmittance reached the maximum value. Similar analyte concentration saturation has also been observed in other studies ^[1-3]^. In addition, as the thickness of the analyte increased, the strength of the surface current of the proposed metasurface was gradually enhanced. This ultimately led to the strength of the anapole resonance generated by the metasurface reaching the maximum value. Then, it no longer underwent significant changes, as shown in Figs. S3(d)-(h). Thus, the anapole resonance excited by the An-graphene-Ms sensor provided a platform for effective control of far-field radiation and near-field enhancement in optics.

**Fig. S3.** (a) The refractive indices of potassium sorbate and sodium benzoate that were experimentally measured to be approximately 1.6. (b) The transmission curve of the An-graphene-Ms sensor that redshifted with increasing the analyte thickness and then saturated. (c) As the analyte thickness increased from 0 (bare) to 24 μm, the amplitude change ∆T increased until it reached saturation. The surface current strength of the proposed metasurface varied with the thickness of the analyte. (d) *t* = 0 μm. (e) *t* = 6 μm. (f) *t* = 12 μm. (g) *t* = 18 μm. (h) *t* = 24 μm.

[1]. S. Tan, F. Yan, W. Wang, H. Zhou, and Y. Hou, "Ultrasensitive sensing with three-dimensional terahertz metamaterial absorber," J. Opt. **20**, 055101 (2018).

[2]. H. Yao, M. Yang, X. Yan, L. Liang, Z. Sun, Q. Yang, T. Wang, X. Hu, Z. Wang, and Z. Li, "Patterned graphene and terahertz metasurface-enabled multidimensional ultra-sensitive flexible biosensors and bio-assisted optical modulation amplification," Results Phys. **40**, 105884 (2022).

[3]. E. Rezagholizadeh, M. Biabanifard, and S. Borzooei, "Analytical design of tunable THz refractive index sensor for TE and TM modes using graphene disks," J. Phys. Appl. Phys. **53**, 295107 (2020).
